# Effect of *SLC16A1* on Hepatic Glucose Metabolism in Newborn and Post-Weaned Holstein Bulls

**DOI:** 10.3389/fgene.2022.811849

**Published:** 2022-05-17

**Authors:** Mingming Xue, Mingkun Song, Duo Yan, Shuaijie Sun, Yadong Wang, Tong Fu, Hanfang Cai, Huifen Xu, Guirong Sun, Kejun Wang, Ming Li

**Affiliations:** College of Animal Science and Technology, Henan Agricultural University, Zhengzhou, China

**Keywords:** liver, RNA-seq, glucose, lactate, SLC16A1, glucose metabolism

## Abstract

**Background:** Patterns of liver energy metabolism significantly differ from birth to adult in cattle undergoing change of rumen rumination. However, the genes involve in hepatic energy metabolism during bovine development and how regulate are still unclear.

**Methods:** In this study, 0-day-old newborn calves (0W) and 9-week-old weaned calves (9W) were used to investigate differences in liver glucose metabolism at these stages of calf development. We did this primarily through the quantitation of energy metabolism indicators, then sequencing the liver transcriptome for each group of claves.

**Results:** The transcriptome results showed 979 differentially expressed genes (DEGs), enriched in animal organ development, catabolic process, transmembrane transport. *SLC16A1* involved in that and was locked to investigate. We explored the effects of *SLC16A1* on glucose and lactate flux *in vitro*. We identified and verified its target, miR-22-3p, through bioinformatics and luciferase reporter assays. Moreover, this study found that miR-22-3p decreased cell activity by negatively regulating the *SLC16A1*. Importantly, our result showed the insulin-induced *SLC16A1* mRNA expression decreased, regulated by promoter activity rather than miR-22-3p.

**Conclusions:** Our study illustrates the role of *SLC16A1* in the liver mediated metabolism of developing calves. These data enrich our knowledge of the regulatory mechanisms of liver mediated glucose metabolism in developing cattle.

## Introduction

### Background

The modes of digestion, absorption, and hepatic metabolism in calves, and other ruminants, undergone tremendous changes from birth to weaning, along with the transition from milk to grain feed as nutritional source. During rumen development, glycogen synthase and glucose-6-phosphatase activity in the liver increases by two-fold (Baldwin and Jesse, 1992), while glucose oxidation decreases (Donkin and Armentano, 1995). Hepatic gluconeogenesis is the primary way lactate is cleared before rumen function is established. Before weaning, sheep utilize lactate/propionic acid for glucose to a much greater degree than adult sheep (Ballard and Oliver, 1965; Savan et al., 1976). In mammalian systems lactate is a major energy source, and also the fulcrum of metabolic regulation (Hui et al., 2017; Brooks, 2020).

Lactate and its transport are essential for the glucose metabolic processes and pH control of all mammalian cells. It is transported through the plasma membrane to cells that use it for gluconeogenesis or as respiratory fuel (Halestrap and Meredith, 2004; Rabinowitz and Enerback, 2020). Monocarbonate transporters (MCTs) are members of the solute carrier family of proteins. MCT1 was the first confirmed member of the MCT family and is encoded by solute carrier family 16 member 1 (*SLC16A1*). *SLC16A1* is responsible for transporting monocarboxylic acid metabolites, such as lactate, pyruvate and ketone bodies (Halestrap and Price, 1999). *SLC16A1* is confirmed to involve in the formation of mitochondrial lactate oxidative complex, explaining the oxidative catabolism of lactate in skeletal muscle cells (Hashimoto et al., 2006). *SLC16A1* is expressed in many tissues, including the liver (Lengacher et al., 2013). Studies confirm the localization of *SLC16A1* in rumen epithelium and the effect of *SLC16A1* on nutrient transport in rumen and intestinal (Muller et al., 2002; Graham et al., 2007; Sivaprakasam et al., 2017). However, it’s unclear the role of *SLC16A1* in hepatic glucose metabolic homeostasis and how to involve in growth and development of ruminants. Remarkably, studies have shown that microRNA is involved in the transcription regulation of *SLC16A1*. miR-342-3p specifically targets *SLC16A1*, which supports the alteration of lactate fluxes, thus disrupting tumor cell metabolic homeostasis (Romero-Cordoba et al., 2018). *SLC16A1* is also targeted by miR-495, which affect levels of insulin, glucose, and liver weight in mice (Liang et al., 2015).

Insulin is one of the main hormones regulating hepatic glucose and lipid metabolism (Khan and Pessin, 2002). In preruminating and ruminating bovines, hormones regulate the hepatic metabolic processes; the sensitivity of the liver to these hormones gradually decreases as the rumen develops (Donkin and Armentano, 1995). Mutations in the promoter of *SLC16A1* lead to excessive secretion of insulin and subsequent hyperinsulinism (Tosur and Jeha, 2017). It is worth considering whether *SLC16A1* gene responds to the regulation of insulin, and then how it regulates liver glucose homeostasis.

In this study, samples were collected in the test based on considering the effects of animal welfare and transportation stress responses (Raza et al., 2021). Then, we identified *SLC16A1* from the differentially expressed genes revealed by transcriptome sequencing of bovine liver tissue from newborn and 9-week-old calves. We found that *SLC16A1* affects intracellular glucose flux by mediating key rate-limiting genes involved in glycolysis and gluconeogenesis. Additionally, we found that miR-22-3p reduced the activity of bovine hepatocytes by negatively regulating *SLC16A1*. Finally, we show that exogenously added insulin inhibits the *SLC16A1* promoter *in vitro*. Altogether, our study revealed a previously unrecognized role of *SLC16A1* in the regulation of hepatic glucose metabolism during the growth of calves.

## Materials and Methods

### Ethics Statement

All experiments and animal care procedures were performed in accordance with protocols and guidelines approved by the Institutional Animal Care and Use Committee (IACUC) of Henan Agriculture University (Zhengzhou, China) (Permit Number: 11-0085; Date: 06-2011).

### Animals and Cells

Six newborn Holstein bull calves, similar in weight, were assigned randomly into two groups (n = 3/group). Calves in the first group were euthanized within 2 h of delivery and had not taken colostrum; these calves were designated 0W (0 weeks of age). The second group of calves were fed according to the “Chinese Beef Cattle Raising Standard” (2004) for 63 days, then were euthanized. These calves were designated 9W (9 weeks of age). During the study, calves were housed in individual pens. On the first day, newborn calves were fed on colostrum. First colostrum was fed within 2 h of birth. From d 2 to 7, raw milk was used to feed calves. Then calves were fed on milk replacer without access to raw milk until weaned at 48 d (Supplementary Table S1). All calves had ad libitum access to starter, starting from 13 d of age to the end of experiment. The composition and nutrient level of the starter diet are described in Supplementary Table S2. In this experiment, decapitation was used to euthanize calves. HEK293T cells and bovine hepatocytes used in this experiment were gifts from Dr. Guirong Sun and Dr. Liqiang Han, respectively.

### Sample Collection

Immediately after sacrifice, jugular venous blood and tissue samples were collected. Blood samples from each calf were stored separately in tubes appropriate for clinical biochemistry analyses. After standing at room temperature for 2 h, serum was collected by centrifugation at 3,000 rpm for 10 min, transferred to fresh tubes, and stored at −80°C. Immediately upon dissection, tissues from the heart, liver, spleen, lung, kidney, rumen, abomasum, duodenum, colon, and dorsal muscle were snap-frozen in liquid nitrogen and stored at −80°C until RNA extraction. Subsamples of liver tissues were taken for section preparation and placed in 4% paraformaldehyde.

### Liver Histology

Liver tissue from six calves was fixed in 4% paraformaldehyde for 24 h. Tissue was then cleared, dehydrated, embedded in paraffin, and cut into 4 µm sections. These sections were subjected to hematoxylin and eosin (HE) staining. Images were collected using a Nikon Eclipse Ti-SR microscope (Tokyo, Japan) and then cell nuclei were analyzed using Image-Pro Plus 6.0 (Media Cybernetics, Houston, TX, United States).

### Biochemical Analysis

Biochemical indices were analyzed according to protocol provide by Changchun Huili Biotech Co. Ltd., Changchun, China. Automatic biochemical analyzer (Chemray240, Rayto, China) was used to measure bovine serum ALT, ALB, ALP, TC, HDL-C, LDL-C, and GLU by Servicebio Biotechnology Co. Ltd. (Wuhan, China). Bovine serum insulin concentration was measured by ELISA (Kejing Biotechnology Co. Ltd., Jiangsu, China). After these tests, cells and cell superstratum culture media were collected for lactate and GLU assays. Lactate concentrations were determined using a lactate assay kit. Intra- and extra-cellular GLU was determined by the glucose oxidase method (Wincey and Marks, 1961) using a GLU Assay Kit. Both kits were purchased from JianCheng Bioengineering Institute, Nanjing, China.

### RNA-Seq and Data Analysis

Total RNA was extracted from liver tissues using an Eastep® Super Total RNA Extraction Kit (Promega, Shanghai, China) according to the manufacturer’s instructions. The integrity of the RNAs was determined using an Agilent 2,100 Bioanalyzer (Agilent, Santa Clara, CA, United States). All samples had an RNA integrity number (RIN) > 8.6 and contained more than 1 μg RNA. Briefly, poly-A mRNA was purified from total RNA using oligo (dT) magnetic beads. Fragmentation was carried out using divalent cations under elevated temperature in an Illumina proprietary fragmentation buffer. After fragmentation, it was used as a substrate for cDNA synthesis and PCR amplification. The concentration and quality of each library was determined using an Agilent 2,100 Bioanalyzer. Index sequences were added to the libraries, and they were combined, diluted to 2 nM, and then a single chain library was generated by alkaline denaturation. A total of six libraries (three libraries each group) were subjected to paired-end 150 bp sequencing using the Illumina HiSeqTM 2,500 platform (Personalbio, Shanghai, China). Group 0W was defined as the control. The raw datasets were deposited in a publicly available database: National Genomics Data Center (NGDC): CRA005376: https://bigd.big.ac.cn/gsa/browse/CRA005376.

Raw reads were filtered using Cutadapt (Martin, 2011) to remove low-quality reads containing 3’ adapter sequences or exhibiting average quality scores less than 20. FastQC (Andrews, 2014) was used to determine quality, content, and quality score distributions of the clean reads. Greater than 80% of the clean reads could be aligned to the bovine reference genome (UMD3.1) using HISAT2. Gene expression levels were standardized as fragments per kilobase of million fragments (FPKM), based on the Reads Count value of each gene obtained from the HTSeq alignment. Differential expression analysis was performed using the R package DESeq (Anders and Huber, 2010). mRNAs with a false discovery rate (FDR)≤0.05 and |log2FoldChange|≥2 were classified as DEGs. A Gene Ontology (GO) enrichment analysis was conducted for the DEGs using TopGO (Alexa and Rahnenführer, 2007). GO terms were grouped into molecular function (MF), biological process (BP), and cell component (CC) classes. DEG functions were inferred using annotations from the Kyoto Encyclopedia of Genes and Genomes (KEGG) database. RNAhybrid and Targetscan were used to predict DEG target miRNAs. Target miRNAs that were recognized by both programs were retained for further analysis. Network interactions among selected DEGs and target miRNAs were constructed and visualized using Cytoscape (Saito et al., 2012).

### RNA Extraction and cDNA Synthesis

Total RNA, from tissue and bovine hepatocytes, was extracted with TransZol (TransGen Biotech, Beijing, China) according to the manufacturer’s protocols. RNA concentration and purity were determined using a Thermo Scientific NanoDropTM 2000 Spectrophotometers and agarose gel electrophoresis. The specific reverse transcribed primers for the miRNA were designed to construct miRNA cDNA libraries according to stem-loop method (Wang, 2009). MiRNA sequences were retrieved from miRBase database (https://mirbase.org/ftp.shtml, Dec. 12, 2020). cDNA was synthesized from 1 μg of RNA using a PrimeScript™ RT reagent kit with gDNA Eraser (TaKaRa, Dalian, China). The cDNA was then used for quantitative real-time PCR (qPCR).

### Quantitative Real-Time PCR

qPCR was used to validate mRNA expression levels determined using high-throughput mRNA-Seq. Eight DEGs were selected for qPCR analysis. Eight DEGs were selected for qPCR analysis including aryl hydrocarbon receptor (*AHR*), cytochrome P450, subfamily I (aromatic compound-inducible), polypeptide 1 (*CYP1A1*), alcohol dehydrogenase 4 (class II), pi polypeptide (*ADH4*), cytochrome P450, family 1, subfamily A, polypeptide 2 (*CYP1A2*), phosphoenolpyruvate carboxykinase 1 (*PCK1*), *SLC16A1*, solute carrier family 16 member 6 (*SLC16A6*) and cytochrome c oxidase assembly factor heme A:farnesyltransferase COX10 (*COX10*). In the function verification section of *SLC16A1*, we detected the relative expression level of genes related to lipid metabolism, including Aconitase 1 (*ACO1*), peroxisome proliferator activated receptor gamma (*PPARG*), diacylglycerol O-acyltransferase 1 (*DGAT1*) and Solute carrier family 2, Member 4 (*SLC2A4*), and glucose metabolism related genes, including *PCK1*, pyruvate carboxylase (*PC*), phosphofructokinase, liver type (*PFKL*) and pyruvate kinase L (*PKLR*). qPCR was performed on each sample for triplicate using SYBER Green and a LightCycler 96 thermocycler (Roche, Indianapolis, IN, United States). The same RNA samples were used for RNA-Seq and qPCR validation. Bovine ribosomal protein S18 (*RPS18*) was used as an internal control for mRNA and miRNA data normalization (Sun et al., 2013). miRNA RT primers and qPCR primers were designed by Biosunya (Biosunya Biotechnology Co. Ltd., Shanghai, China) and listed in Supplementary Table S3. The 2^–∆∆Ct^ method was used to determine relative mRNA and miRNA abundance (Livak and Schmittgen, 2001).

### Western Blot

Samples were harvested on ice using RIPA buffer (EpiZyme, Shanghai, China) with 1% Protease Inhibitor Cocktail (New Cell & Molecular Biotech Co. Ltd., Suzhou, China). Protein concentrations were measured by BCA protein assay. PAGE gels were prepared with a 15% PAGE Gel Fast Preparation Kit (EpiZyme, Shanghai, China) according to the manufacturer’s protocols. 20 μg total protein from each sample was subjected to protein denaturation, resolved on a 15% SDS-PAGE gel, then transferred onto a PVDF membrane (Millipore). Membranes were pre-treated with blocking buffer containing 5% milk, then incubated with primary antibody overnight at 4°C. After washing with TBST six times (5 min each), membranes were incubated with horseradish peroxidase-conjugated secondary antibody (EARTHOX, Shanghai, China) for 2 h at room temperature. The membranes were washed six times in TBST, then incubated with ECL reagent to visualize protein bands. The primary antibody MCT1 (1:4,000 dilution) was purchased from Abcam. β-actin (1:10,000 dilution) was purchased from Bioworld (Nanjing, China). Western blots were scanned, and the images were quantified using ImageJ (Schindelin et al., 2015).

### Plasmid Construction and Adenovirues Package

The 3’ untranslated region (UTR) of the bovine *SLC16A1* gene, containing the miR-22-3p binding site, was amplified by PCR using bovine genomic DNA as the template. The amplicon was purified then ligated into the *XhoI*/*NotI* site of the psiCHECK-2 vector. The final recombinant plasmid was designated psiCHECK2-*SLC16A1* WT. Using site-directed mutagenesis in the seed region of the miR-22-3p site, a mutant *SLC16A1* 3’UTR reporter was constructed (psiCHECK2-*SLC16A1* MUT). The promoter region of the bovine *SLC16A1* was amplified and then subcloned into the *KpnI*/*XhoI* site of the pGL4.10 Luciferase Vector (Promega). Coding sequence of bovine *SLC16A1* was amplified and then subcloned into the necessary adeno expression vectors for viral production. Adenovirues were generated by VectorBuilder. All plasmids were purified using an EndoFree Mini Plasmid Kit II (TIANGEN, Beijing, China) then sequenced by Biosunya Biotechnology Co. Ltd. (Shanghai, China). Primers are listed in Supplementary Table S4.

### Cell Transfection and Reporter Assay

Cells were seeded in 24-well plates and cultured until approximately 70% confluent. RNA fragments, including *SLC16A1* siRNA, miR-22-3p, and negative control (NC), were transfected into bovine hepatocytes using Lipofectamine 3,000 (Invitrogen, California, United States) following the manufacturer’s instructions, and cultured for 48 h. Luciferase reporter experiments were performed in HEK293T cells. PsiCHECK2-*SLC16A1* WT or psiCHECK2-*SLC16A1* MUT was co-transfected with NC or a miR-22-3p mimic (GenePharma, Shanghai, China) using Lipofectamine 2000 according to the manufacturer’s instructions. The medium was replaced after 6 h. Transfections were performed using 800 ng luciferase reporter and 80 ng of internal control plasmid pRL-TK (Promega). Relative luciferase activity was measured using the Dual-Luciferase Reporter Assay System (Promega) and a Synergy LX microplate reader (BioTek, Beijing, China). Bovine hepatocytes were seeded in 6-well plates and cultured until approximately 40% confluent. Bovine hepatocytes were infected by recombinant adenovirus at 100 multiplicity of infection for 48 h.

### Cell Viability Assay

1×10^4^ bovine hepatocytes were seeded into 96-well plates and cultured until approximately 70% confluent. Cells were transfected with a negative control (NC), a miR-22-3p mimic, or *SLC16A1* siRNA using Lipofectamine 3,000, following the manufacturer’s protocol. At 0, 24, 48, and 72 h post-transfection, cell viability was assessed using a Cell Counting Kit-8 (CCK-8) colorimetric assay (Kemix). Briefly, 10 µl of CCK-8 solution was added to each well and incubated for 2 h. Absorbance was measured at 450 nm using a BioTek microplate reader. Five biological replicates were measured for each group.

### Cell Culture and Exogenous Insulin Treatment

Bovine hepatocytes were maintained in high glucose medium supplemented with 10% fetal bovine serum (Biological Industries, Israel). Bovine hepatocytes were treated with 0.1 μM biosynthetic human insulin (Novolin R, Brazil) for 24 h after 12 h of starvation. Transfected cells were treated with 0.1 μM recombinant human insulin for 24 h before lysis and analysis of luciferase activity.

### Statistical Analysis

Statistical analyses were performed using SPSS18.0 (IBM, Chicago, IL, United States) *p < 0.05; **p < 0.01. Student’s *t* test was used to compare 2 groups of samples, and a one-way ANOVA was used to compare more than two groups. All data are expressed as the mean ± SEM. Pearson’s correlation analysis was conducted in order to determine the linear correlation of fold changes calculated with RNA-Seq vs qPCR data. The same statistical method was used to calculate the correlation between genes and phenotypes.

## Results and Discussion

### Energy Metabolism Indicators and Liver Histology

**FIGURE 1:**
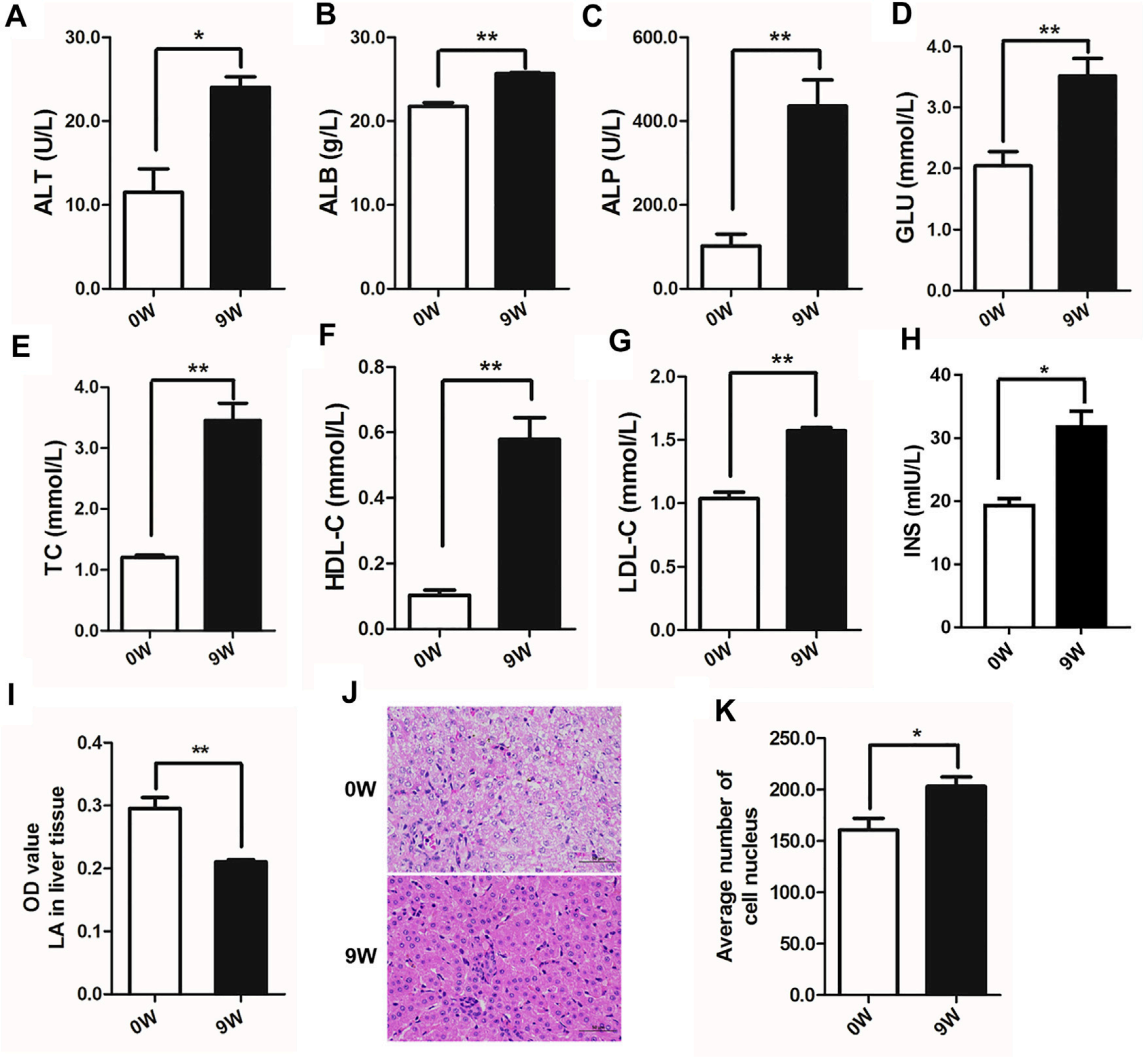
Serum indices, hepatic lactate and histological observation of calf liver tissues from 0W and 9W animals. **(A)** alanine aminotransferase (ALT); **(B)** albumin (ALB); **(C)** alkaline phosphatase (ALP); **(D)** glucose (GLU); **(E)** total cholesterol (TC); **(F)** high-density lipoprotein cholesterol (HDL-C); **(G)** low-density lipoprotein cholesterol (LDL-C); **(H)** insulin; **(I)** lactate (LA) levels in liver tissues; **(J)** HE stained liver sections; **(K)** Quantification of the number of cell nuclei in liver sections.

Pattern of liver energy metabolism significantly differed from birth to adult in cattle mainly resulting from rumen rumination which usually beginning at 2–4 weeks of age (Swanson and Harris, 1958). In this study, indicators of energy metabolism were compared between 0-day-old newborn (0W) and 9-week-old weaned (9W) male Holstein calves (Figure 1). Seven biochemical indicators in venous blood, including serum alanine aminotransferase (ALT), albumin (ALB), alkaline phosphatase (ALP), glucose (GLU), total cholesterol (TC), high-density lipoprotein cholesterol (HDL-C), and low-density lipoprotein cholesterol (LDL-C), showed significantly higher level in 9W calves than that of 0W calves (*p* < 0.05) (Figures 1A–G). We also observed that 9W calves had significantly higher serum insulin levels and significantly lower hepatic lactate levels than 0W calves (Figures 1H,I). These indicated that ability of glucose and lipid metabolism in 9W calves was significantly altered compared to 0W calves. Liver tissue histology revealed that liver tissue from 9W calves was dense and contained hepatocytes with clear nucleoli and abundant cytoplasm, compared to 0W calves (Figures 1J,K).

### Liver Transcriptomic Analysis and Identification of DEGs

**FIGURE 2:**
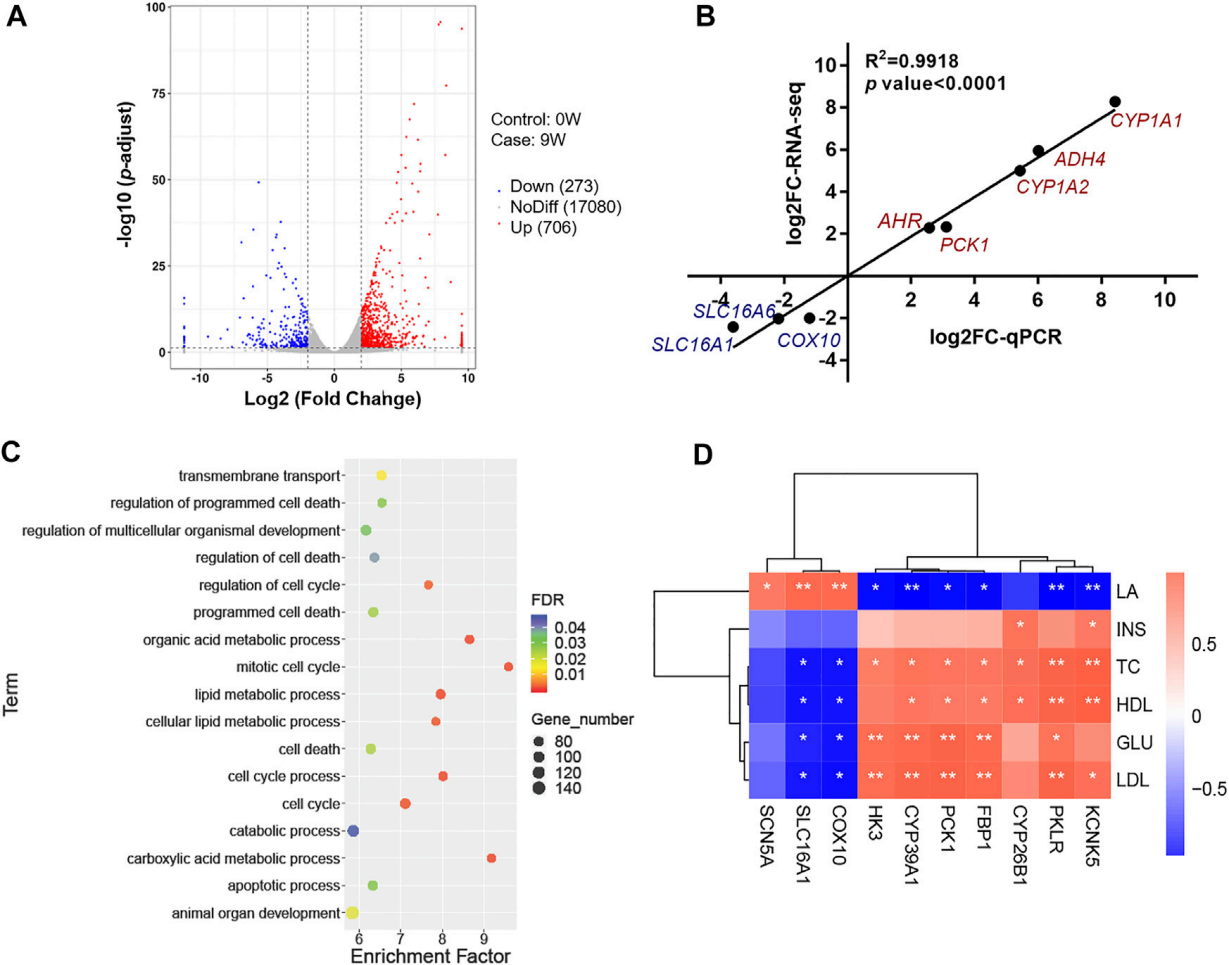
Analysis and verification of RNA sequencing data. **(A)** Volcano plot showing expressed genes and criteria for DEG classification; **(B)** Analysis of the correlation between RNA-seq and qPCR; **(C)** Functional enrichment analyses of DEGs; **(D)** Correlation analysis between DEGs transcription levels and phenotypic traits.

To uncover the potential molecular mechanism involving the difference of liver development and metabolism, six cDNA libraries were constructed from the livers of 0W and 9W calves using transcriptome sequences. Reads per library ranged from 42.55 million to 50.83 million. Over 90% of reads exhibited quality (Q) scores of 30. High-quality reads from each library were aligned to the reference genome using Hisat2 software (Supplementary Table S5). Expression profile from libraries within the same group were strongly correlated (at least 0.94; Supplementary Figure S1A). The result showed great homogeneity within each group, while it still no ignored the limitations of a minimum sample size. A total of 18059 genes were expressed in liver tissues from both groups. We identified 979 DEGs, of which 706 were relatively more abundant and 273 were less abundant in the 9W group relative to the 0W group (Figure 2A and Supplementary Table S6). To validate these expression levels, we performed qPCR on 8 DEGs (Supplementary Figure S1B and Supplementary Table S7). The qPCR results were consistent with the transcriptome sequencing results (Pearson’s correlation coefficient = 0.99, Figure 2B), underscoring the reliability of the transcriptome data.

### Functional Enrichment Analysis of DEGs

DEGs were subjected to Gene Ontology (GO) and KEGG pathway analyses. GO terms and KEGG pathways with the greatest significant enrichment are shown in Supplementary Figure S2A, S1B. Result showed GO enriched terms included “plasma membrane part”, “catalytic activity”, and “cell activation” (Supplementary Figure S2A and Supplementary Table S8). KEGG pathways included metabolism of xenobiotics by cytochrome P450, retinol metabolism, steroid hormone biosynthesis, and glutathione metabolism were clustered by significantly DEGs (Supplementary Figure S2B and Supplementary Table S9). To better understand the function of genes involved in hepatic development and metabolism, this study focused on these DEGs between 0W and 9W calves were generally involved in cell functions such as cell death and cell cycle, and energy metabolism processes such as transmembrane transport, carboxylic acid metabolic process, and cellular lipid metabolic process (Figure 2C and Supplementary Table S1). To identify these potential DEGs, correlations were examined between 10 DEGs associated with hepatic energy metabolism and phenotypic data. Seven DEGs, including pyruvate kinase L/R (*PKLR*), hexokinase 3 (*HK3*), phosphoenolpyruvate carboxykinase 1 (*PCK1*), cytochrome P450, family 39, subfamily A, polypeptide 1 (*CYP39A1*), fructose-bisphosphatase 1 (*FBP1*), cytochrome P450, family 26, subfamily B, polypeptide 1 (*CYP26B1*) and potassium two pore domain channel subfamily K member 5 (*KCNK5*), showed a positive correlation to INS, TC, HDL, GLU and LDL, while negative correlation to LA level. Other three DEGs, including *SLC16A1*, cytochrome c oxidase assembly factor heme A: farnesyltransferase COX10 (*COX10*) and sodium voltage-gated channel alpha subunit 5 (*SCN5A*), showed converse correlation pattern (Figure 2D). These DEGs are essential for lipid metabolism (Matsuoka et al., 2020), mitochondrial function (Liu et al., 2019), energy metabolism (Wang et al., 2019), and organ development (Alvarez-Baron et al., 2011; Daniel et al., 2020; Sokolov et al., 2020). Notably, *SLC16A1* was observed involving in transmembrane transport and showed the highest positive correlation to LA (Figure 2D). *SLC16A1* encodes monocarboxylate transporter MCT1, which is responsible for LA exchange of cell (Halestrap, 2013b).

### Effect of SLC16A1 on Hepatic Glucose Metabolism

In this study, we observed that *SLC16A1* mRNA level were negatively correlated to HDL, LDL, and TC content (Figure 2D). To further examine the effect of *SLC16A1* on hepatic lipid metabolism, we investigated lipid metabolism related genes in bovine hepatocytes when *SLC16A1* was silenced (Supplementary Figure S3A). We found that disrupting *SLC16A1* did not alter the expression of *ACO1*, *DGAT1*, *PPARG* and *SLC12A4* genes (Supplementary Figure S4A–D). This is consistent with result that expression of lipid metabolism related genes was not altered in liver of *SLC16A1* ^+/−^ mice relative to wildtype mice when feeding normal chow (Lengacher et al., 2013).

**FIGURE 3:**
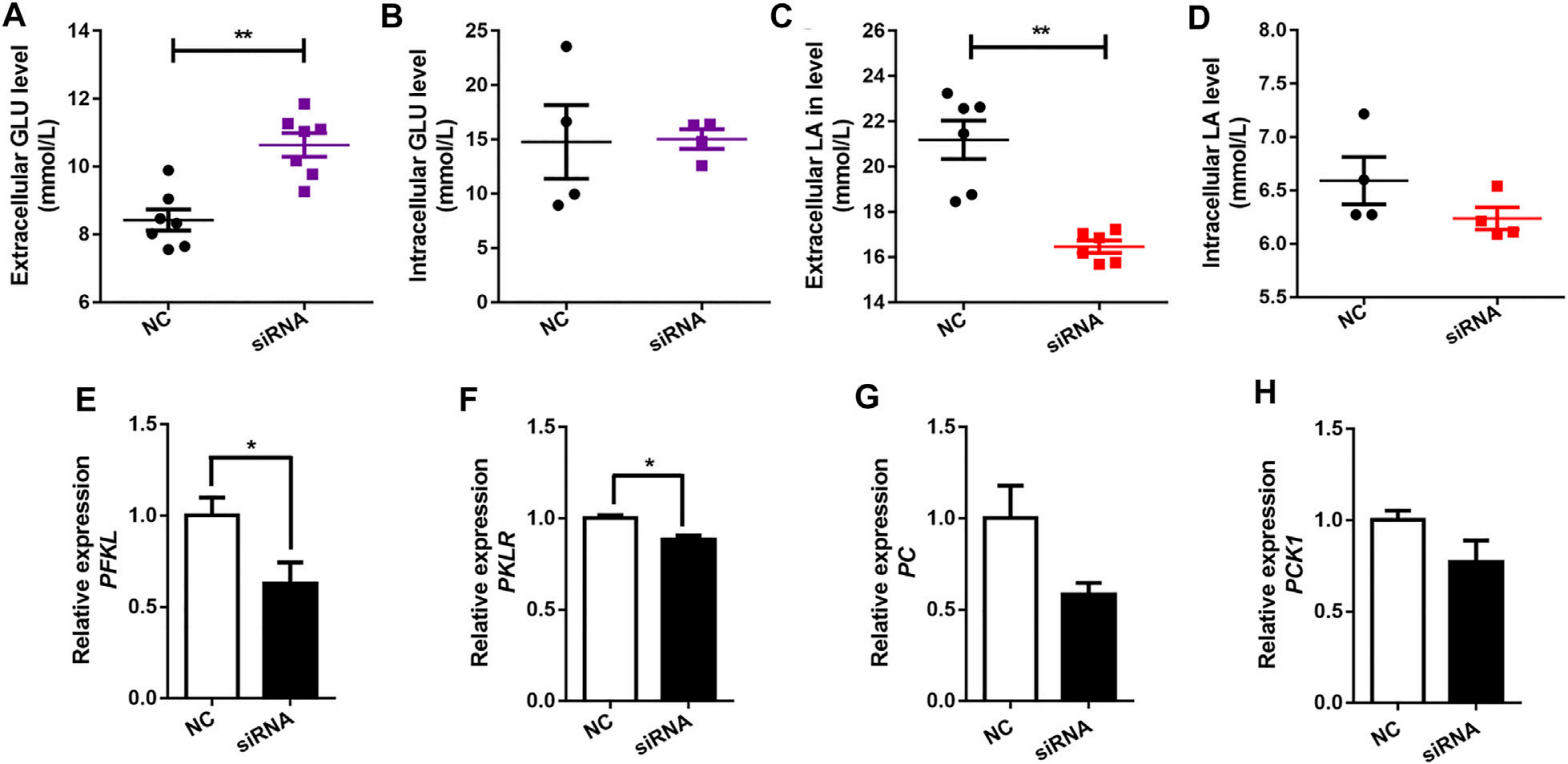
Effect of *SLC16A1* silencing on glucose, lactate flux and glucose metabolism related genes in bovine hepatocytes. **(A)** Extracellular and **(B)** intracellular GLU levels; **(C)** Extracellular and **(D)** intracellular LA levels. **(E)** *PFKL*, **(F)** *PKLR*, glycolysis; **(G)** *PC*, **(H)** *PCK1*, gluconeogenesis. siRNA, *SLC16A1*; NC, negative control siRNA.

Pearson’s correlation analysis showed that the expression of the *SLC16A1* was negatively correlated with serum glucose levels, and positively correlated with hepatic lactate level (Figure 2D). A crucial role of *SLC16A1* in the regulation of energy provision relies on lactate produced by glucose metabolism (Sun et al., 2013; Faubert et al., 2017). Lactate is a product of glycolysis and a gluconeogenic precursor (Brooks, 2007; 2009; Emhoff et al., 2013). *SLC16A1* is an important factor mediating lactate transport (Halestrap, 2013a). Subsequently, we investigated whether glucose metabolism could be influenced in bovine hepatocytes when *SLC16A1* was silenced. Higher extracellular glucose level was observed in *SLC16A1* silenced bovine hepatocytes (Figure 3A), while intracellular glucose level was not altered (Figure 3B). We also observed that extracellular lactate level decreased in *SLC16A1* silenced cells (Figure 3C), and without change for intracellular (Figure 3D). Disrupting *SLC16A1* function leaded to a decrease in glycolysis resulting from the reducing of *PFKL* and *PKLR* expression (Figures 3E,F), which are the key glycolysis rate-limiting enzymes, while there was no significant difference in transcription of *PC* and *PCK1* (Figures 3G,H). These results suggest that decreasing of glycolysis process induced by *SLC16A1* silence could suppress the generating of lactate leading to reduce lactate flux release, and possibly maintain homeostasis of intracellular lactate (Le Floch et al., 2011; Doherty et al., 2014). On the other side, decreasing of glycolysis induced by *SLC16A1* silence might suppresses utilization of glucose leading to possibly reduce extracellular glucose uptake (Doherty et al., 2014). Low levels of glucose transport and glycolysis reflect a decrease in cell anabolism, which hinders cell proliferation (Vander Heiden et al., 2009). These events might converge to influence cell viability in *SLC16A1* silenced hepatocytes (see below, Figure 6E).

**FIGURE 4:**
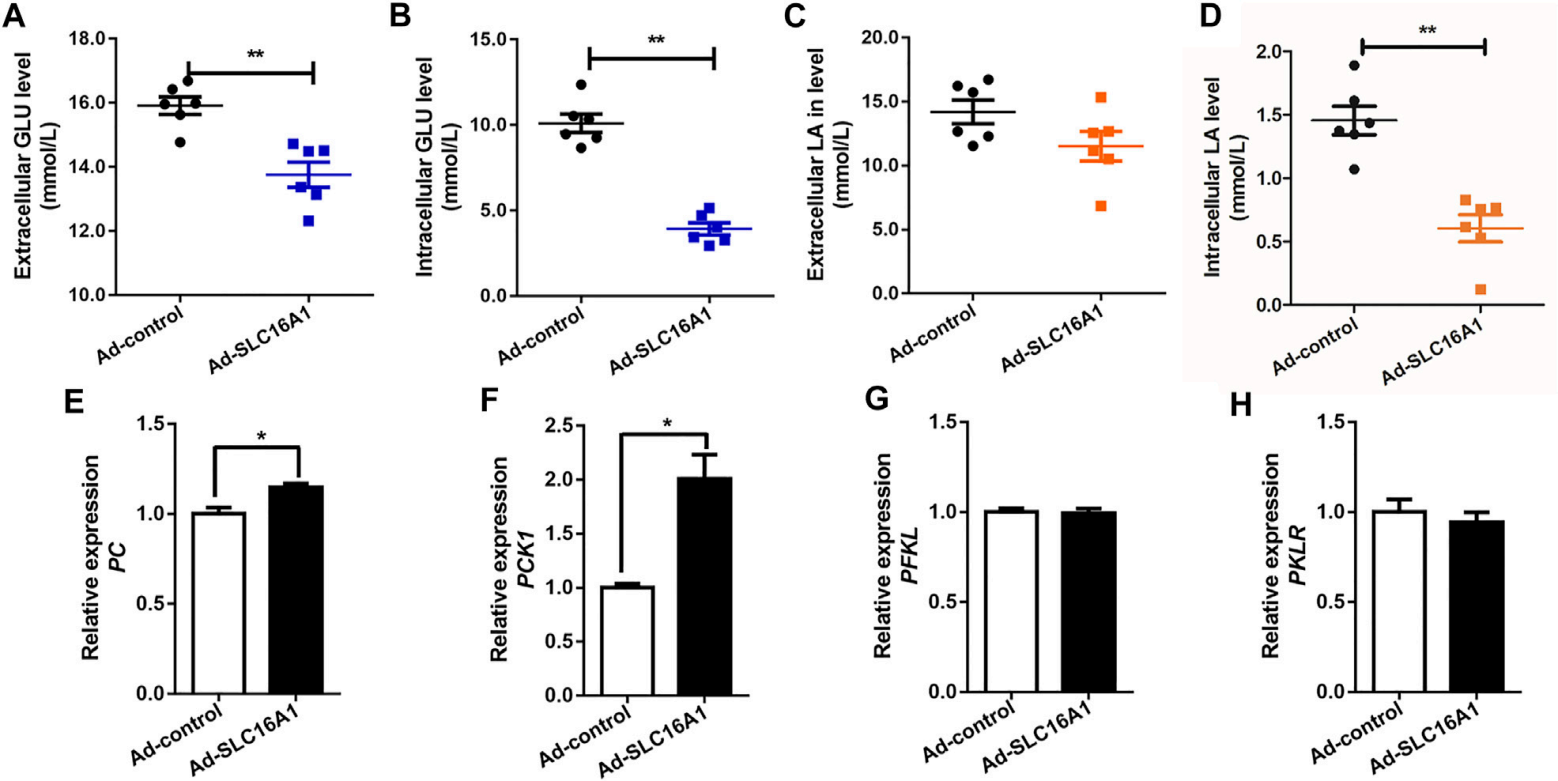
Effect of *SLC16A1* overexpressing on glucose, lactate flux and glucose metabolism related genes in bovine hepatocytes. **(A)** Extracellular and **(B)** intracellular GLU levels; **(C)** Extracellular and **(D)** intracellular LA levels. **(G)** *PC*, **(H)** *PCK1*, gluconeogenesis; **(E)** *PFKL*, **(F)** *PKLR*, glycolysis. Ad-control, empty vector adenovirus; Ad-*SLC16A1*, *SLC16A1* recombinant vector adenovirus.

We also created *SLC16A1* overexpression bovine hepatocytes model (Supplementary Figure S3B). Lower extracellular and intracellular glucose levels was observed in *SLC16A1* overexpression group (Figures 4A,B). In this condition, we presumed that more glucose was taken up to remain glucose homeostasis as fuel for TCA cycle. Results show the expression levels of *PC* and *PCK1*, which are the key gluconeogenic rate-limiting enzymes, increased significantly with overexpression of *SLC16A1* in bovine hepatocytes (Figures 4E,F), while there was no significant difference in transcription of *PFKL* and *PKLR* (Figures 4G,H). The activation of *PC* means that the metabolic capacity of TCA cycle is enhanced (Liu et al., 2020). Glycolysis was activated to balance energy homeostasis in hepatocytes. Lactate is gluconeogenic substrates and reported the major energy source for most tissues and cells (Hui et al., 2017; Ippolito et al., 2019). Lactate was consumed as a fuel energy source and gluconeogenic precursor, and intracellular lactate level decreased in *SLC16A1* overexpression cells (Figure 4D), and without change for extracellular (Figure 4C). Our results supported the initial hypothesis. These suggested that glucose metabolism in *SLC16A1* overexpressing bovine hepatocytes was accelerated with increasing glucose flux, as well as a positive response was provided by lactate gluconeogenesis.

### miR-22-3p Binds SLC16A1 Inhibiting Bovine Hepatocytes Activity

**FIGURE 5:**
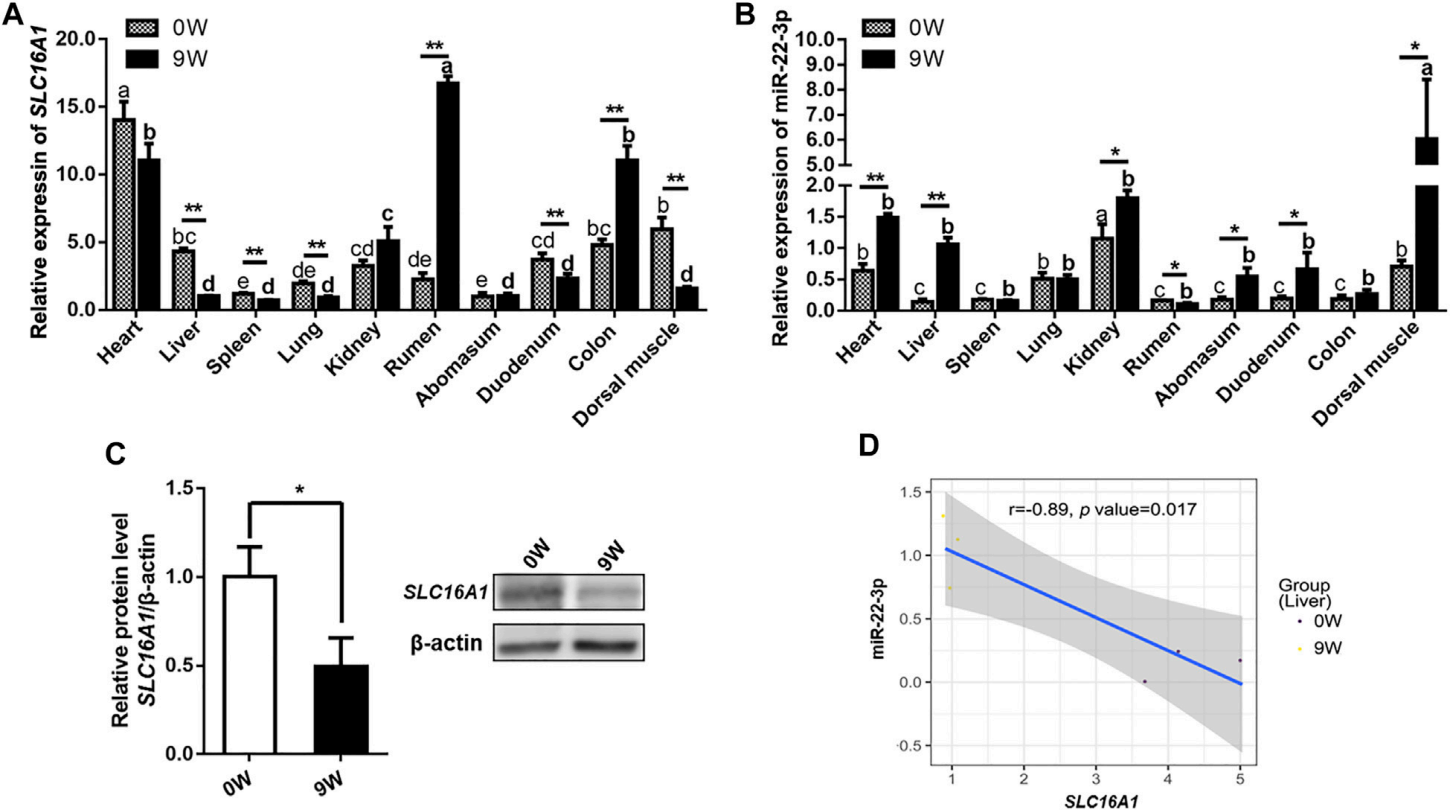
Validation of the expression patterns of bovine *SLC16A1* and miR-22-3p. **(A)** Relative expression of *SLC16A1* and **(B)** miR-22-3p in ten investigated tissues from 0W and 9W calves. **(C)** Relative expression of *SLC16A1* in bovine liver tissues determined by western blotting. **(D)** Pearson correlation analysis of *SLC16A1* and miR-22-3p expression in bovine liver tissues.

We all know that epigenetic factors, such as miRNA, function on gene expression profile since genetic basis could not alter during liver development. The potential target relationship between miR-22-3p and *SLC16A1* was predicted by Targetscan (Agarwal et al., 2015) and RNAhybrid (Jan and Marc, 2006). Then, we compared the expression levels of miR-22-3p and *SLC16A1* in ten investigated tissues from 0W and 9W calves. First, we constructed the tissues expression profile of bovine *SLC16A1*. The relative expression level of *SLC16A1* in most tissues of group 9W was significantly lower than that of group 0W except rumen and colon (Figure 5A). With the gradual improvement of gastrointestinal function, the expression levels of *SLC16A1* in rumen and colon tissues of 9W calves was significantly increased, which was closely related to the nutrient transport of bovine gastrointestinal by *SLC16A1* (MCT1) (Arroyo et al., 2017; Sivaprakasam et al., 2017). Then, we compared the relative expression level of miR-22-3p in the same tissues, and results showed converse expression pattern between 0W and 9W group was observed in almost all investigated tissues (Figures 5A,B). The mRNA and protein levels of *SLC16A1* were lower in liver of 9W calves than that of 0W (Figures 5A,C), while miR-22-3p expression was lower in liver of 0W than that of 9W (Figure 5B). Pearson correlation analysis supported the significant negative correlation between *SLC16A1* and miR-22-3p levels (Figure 5D).

**FIGURE 6:**
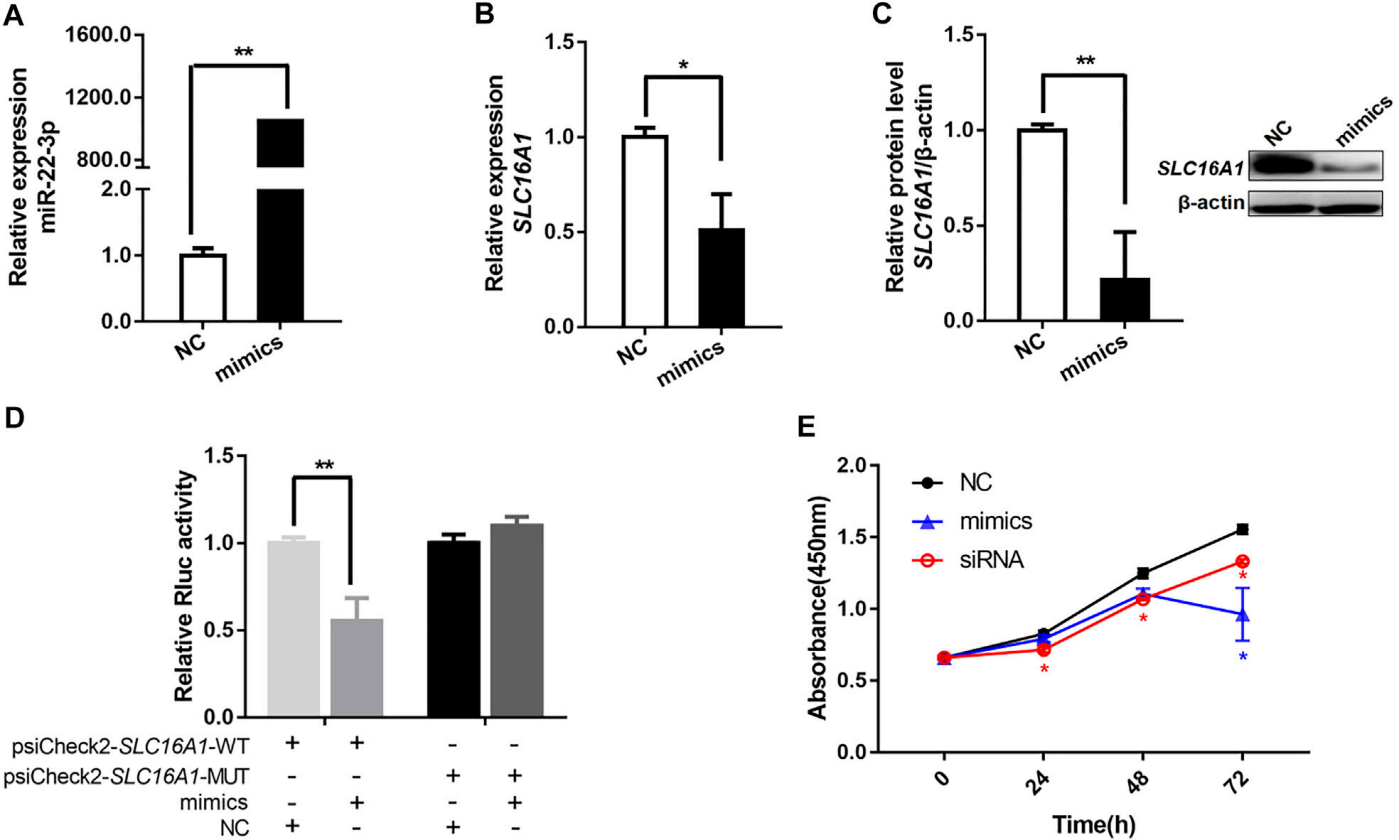
miR-22-3p inhibits bovine hepatocytes activity by targeting the 3’UTR of *SLC16A1*. In bovine hepatocytes transfected with miR-22-3p mimic, **(A)** relative expression of miR-22-3p, and **(B)** relative expression of *SLC16A1* mRNA and **(C)** protein. **(D)** Inhibitory effect of miR-22-3p on *SLC16A1* 3’UTR, determined using a dual-luciferase assay to compare miR-22-3p target sites in the *SLC16A1* mRNA 3’ UTR vs a mutant variant. **(E)** Bovine hepatocytes activity determined by CCK-8 assay after transfection with miR-22-3p mimic or *SLC16A1* siRNA.

This implies a potential targeting of miR-22-3p on *SLC16A1* gene. Using a miR-22-3p mimic, we overexpressed miR-22-3p in bovine primary hepatocytes (Figure 6A). Consistent with the previous results, *SLC16A1* mRNA and protein expression levels decreased significantly compared to cells transfected with the negative control (Figures 6B,C). Direct binding between miR-22-3p and *SLC16A1* located at the 830-835 bp within the 3’UTR was confirmed in a luciferase assay. MiR-22-3p significantly reduced the relative Rluc activity of the wild-type *SLC16A1* reporter vector, but not of the mutated *SLC16A1* reporter vector that contained point mutations in the seed region of the miR-22-3p binding site (Figure 6D). These data demonstrate that *SLC16A1* is the direct target gene of miR-22-3p. Previous studies demonstrate miR-22-3p not only results in apoptosis of cardiomyocytes (Zhou et al., 2017), but also affects cell proliferation in hepatocellular carcinoma (Chen et al., 2016). *SLC16A1* regulates the cell survival in lung cancer cells (Fritsche-Guenther et al., 2018). To explore the role of *SLC16A1* in the proliferation of bovine hepatocytes, cells were transfected with *SLC16A1* siRNA or miR-22-3p mimic and cultured for 72 h. Growth of the *SLC16A1* silenced cells was lower than the control group. Growth of the mimic-transfected cells was comparable to the *SLC16A1-*silenced cells until 48 hpt, at which time mimic-transfected cell growth slowed dramatically (Figure 6E). These results suggest that miR-22-3p reduces the activity of bovine hepatocytes by negatively regulating *SLC16A1*. A gene is usually regulated by multiple miRNAs. We do not rule out that other target miRNAs may influence hepatocyte activity by regulating *SLC16A1*.

### Insulin Inhibits the Expression of SLC16A1 by Promoter Activity Rather Than miRNA-22-3p

**FIGURE 7:**
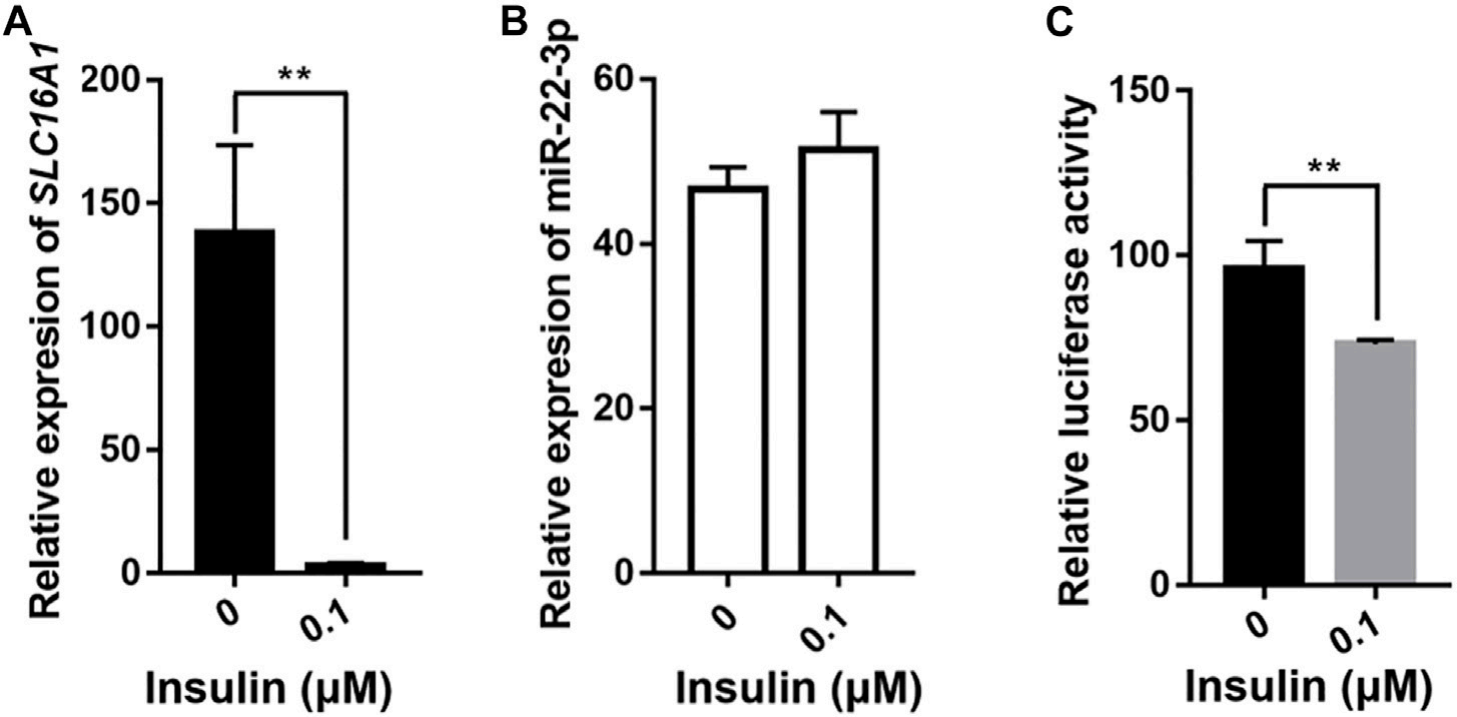
Effect of insulin on expression of *SLC16A1* and miR-22-3p. QPCR assays of **(A)** *SLC16A1* and **(B)** miR-22-3p expression in transfected bovine hepatocytes treated with 0.1 μM insulin for 24 h after 12 h of starvation. **(C)** Dual-luciferase assay of bovine *SLC16A1* promoter activity. n = 5 in each group.

*SLC16A1* is closely associated with diseases characterized by inappropriate insulin secretion, such as type II diabetes (T2D) (Rutter et al., 2015; Higuchi et al., 2020) and exercise-induced hyperinsulinism (EIHI) (Otonkoski et al., 2007; Marquard et al., 2013). In mice, hyperglycemia and hyperinsulinemia can be alleviated by transplantation of stem cells expressing suppress of *SLC16A1* (Liang et al., 2015). We also observed negative correlation between *SLC16A1* mRNA level and insulin content (Figure 2D). To investigate whether insulin influences *SLC16A1* expression in bovine and whether it is regulated by miR-22-3p, we treated bovine hepatocytes with 0.1 μM insulin for 24 h after 12 h of starvation. The relative expression level of *SLC16A1* was significantly reduced in insulin-stimulated bovine hepatocytes, but it did not affect the expression of miR-22-3p (Figures 7A,B). Insulin can regulate gene expression, such as insulin like growth factor binding protein 1 (*IGFBP-1*) and phosphoenolpyruvate carboxykinase (*PEPCK*), by stimulating promoter activity (Gan et al., 2005; Yeagley and Quinn, 2005). Using the luciferase reporter system, we sought to determine whether the insulin-dependent depression of *SLC16A1* expression was mediated by *SLC16A1* promoter activity. After cells were incubated with 0.1 μΜ insulin for 24 h, promoter activity was significantly reduced relative to the control (Figure 7C). Our results showed low expression of *SLC16A1* induced by insulin was due to the inhibition of the *SLC16A1* promoter activity (Figures 7A,C), rather than the negative regulation of miR-22-3p (Figure 7B). Further studies were required to uncover upstream regulators and the molecular pathway of insulin affecting the *SLC16A1* promoter activity.

## Conclusion

*SLC16A1* is an important molecule of hepatic metabolism in calf development ages. It acts as an intermediate hub for insulin, glucose, and lactic acid to regulate energy homeostasis in bovine hepatocytes. Insulin-induced *SLC16A1* effected glucose flux and maintained energy homeostasis in bovine hepatocytes by regulating gluconeogenesis or glycolysis. The transporter *SLC16A1* plays an important role in maintaining glucose metabolism and growth of bovine hepatocytes.

## Data Availability

The datasets presented in this study can be found in online repositories. The names of the repository/repositories and accession number(s) can be found below: https://bigd.big.ac.cn/gsa/browse/CRA005376.

## Supplementary Material

Supplementary Figure S1 Correlation analysis of gene expression level. **(A)** Correlation of expression level among libraries; **(B)** Validation of DEG expression level by qPCR.

Supplementary Figure S2 Top greatest significantly enriched GO and KEGG cluster. **(A)** GO terms and **(B)** KEGG pathways.

Supplementary Figure S3 Efficiency of SLC16A1 knock-down or overexpression. SLC16A1 relative expression levels in SLC16A1 **(A)** silencing or **(B)** overexpressing bovine hepatocytes.

Supplementary Figure S4 Effect of SLC16A1 silencing on transcription of genes related to lipid metabolism. **(A)** ACO1, lipid oxidation; **(B)** PPARG, metabolism; **(C)** DGAT1, synthesis; **(D)** SLC2A4.
