# Supplementary figures and images for: Effect of SLC16A1 on Hepatic Glucose Metabolism in Newborn and Post-Weaned Holstein Bulls
Source: Front Genet. 2022 May 17;13:811849. doi: 10.3389/fgene.2022.811849 (PMC9156795; doi:10.3389/fgene.2022.811849)

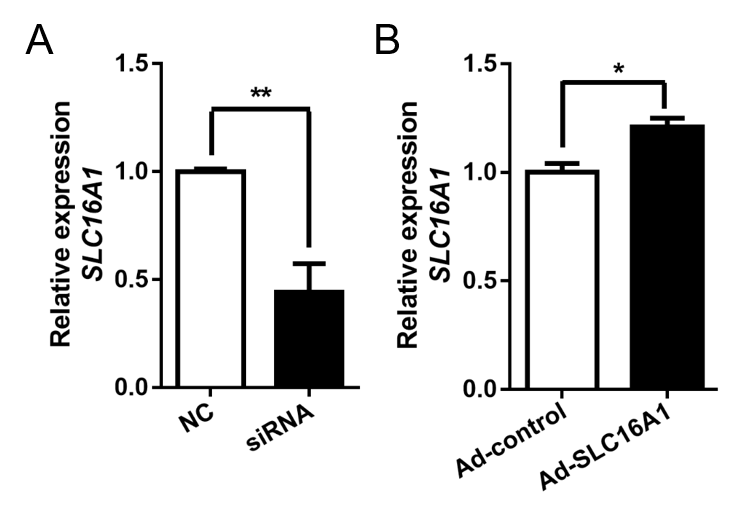

Supplement: Supplementary file 4 [file Image3.TIF]

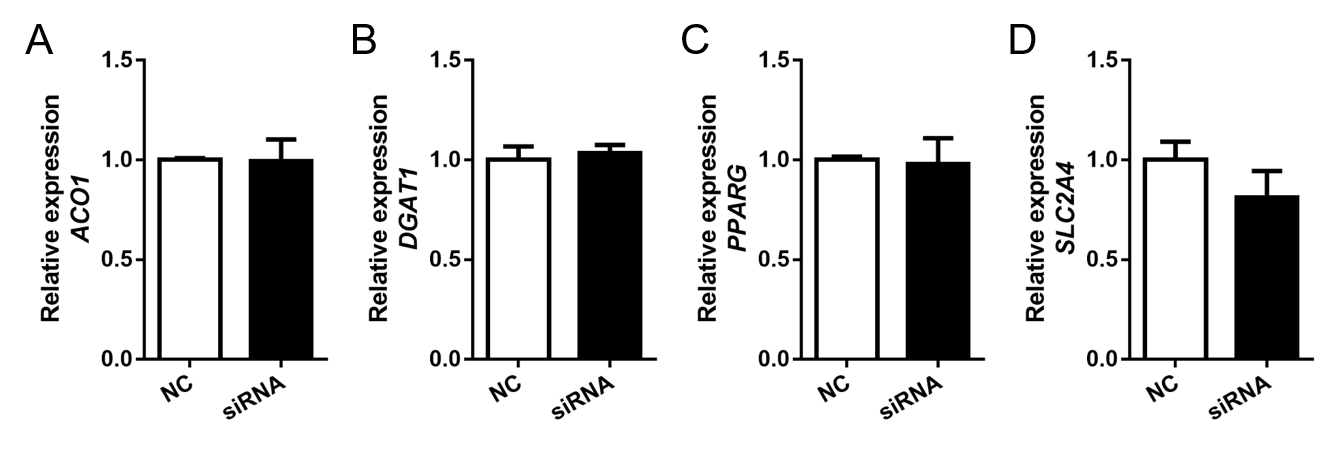

Supplement: Supplementary file 5 [file Image4.TIF]

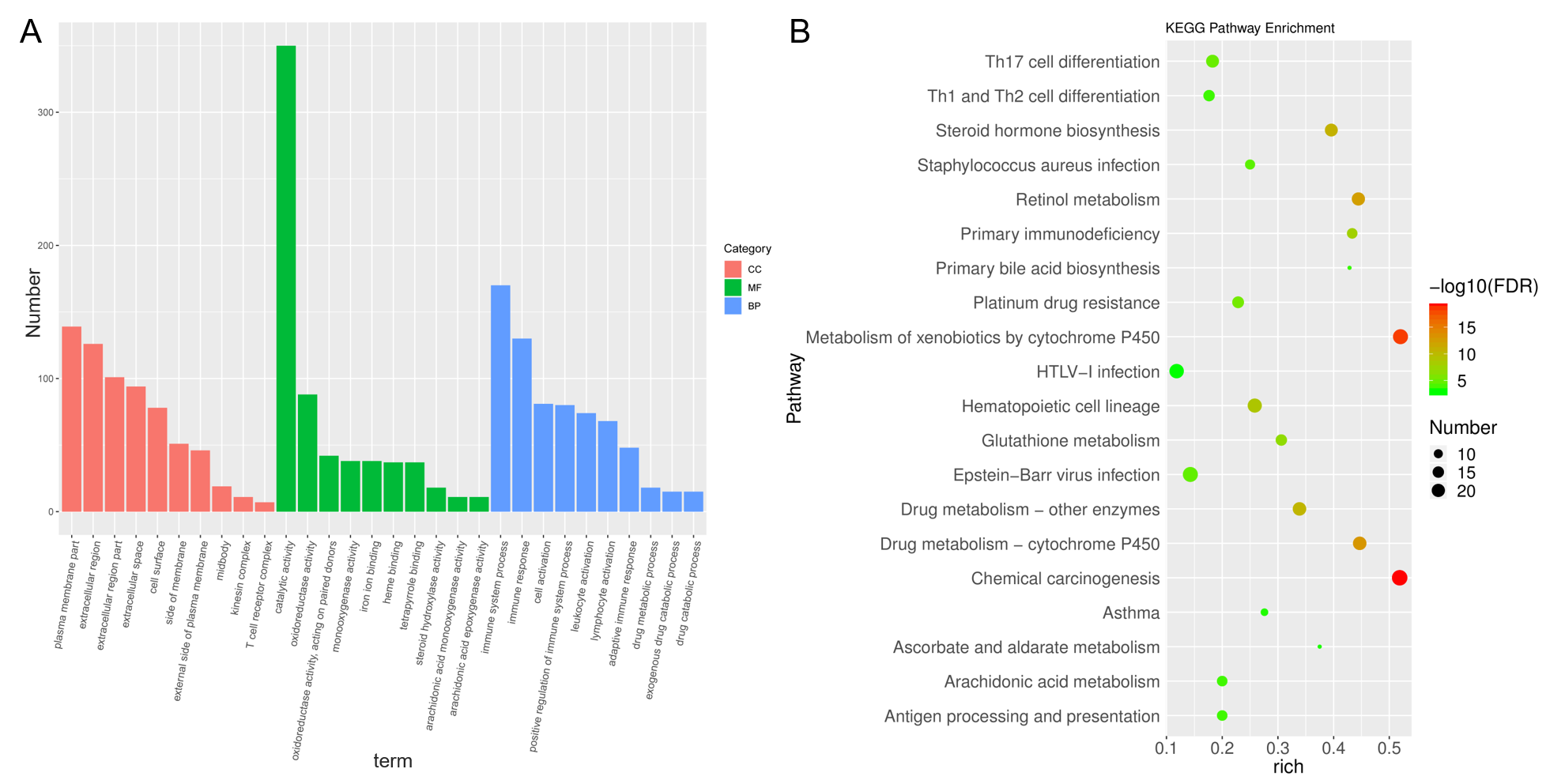

Supplement: Supplementary file 6 [file Image2.TIF]

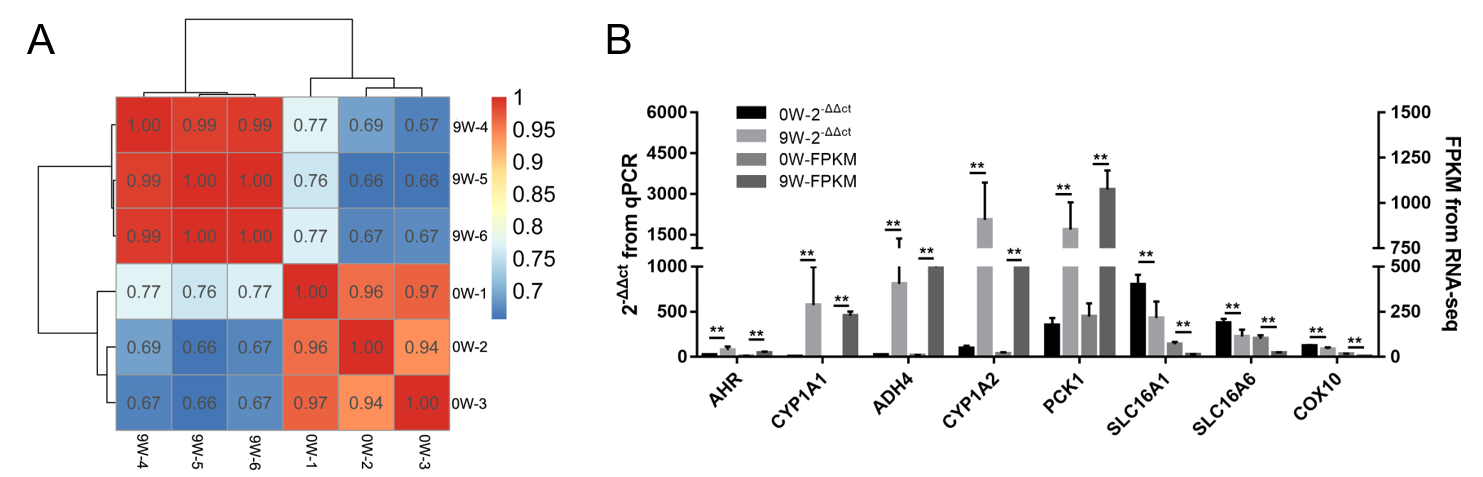

Supplement: Supplementary file 7 [file Image1.TIF]
