# Supplementary material for: Effect of SLC16A1 on Hepatic Glucose Metabolism in Newborn and Post-Weaned Holstein Bulls
Source: Front Genet. 2022 May 17;13:811849. doi: 10.3389/fgene.2022.811849 (PMC9156795; doi:10.3389/fgene.2022.811849)
Supplement: Supplementary file 11 [file Table11.DOCX]

https://www.jianguoyun.com/p/DYiSkgcQi8mDChj_zZwE
